# EDSS and infratentorial white matter lesion volume are considered predictors of fatigue severity in RRMS

**DOI:** 10.1038/s41598-023-38368-3

**Published:** 2023-07-14

**Authors:** Mohammed Y. Ezzeldin, Doaa M. Mahmoud, Shady M. Safwat, Radwa Kamel Soliman, Tarek Desoky, Eman M. Khedr

**Affiliations:** 1 Neuropsychiatric Department, Faculty of Medicine, South Valley University, Qena University, Qena, Egypt; 2 Department of Neuropsychiatry, Faculty of Medicine, Assiut University Hospital, Assiut, Egypt; 3 Radiology Department, Faculty of Medicine, Assiut University, Assiut, Egypt; 4 Neuropsychiatric Department, Faculty of Medicine, Aswan University, Aswan, Egypt

**Keywords:** Medical research, Neurology, Pathogenesis, Risk factors, Signs and symptoms

## Abstract

Fatigue is a common disabling symptom of relapsing remitting multiple sclerosis (RRMS). Many studies have linked grey matter atrophy to fatigue, but white matter lesion load (WM-LL) has received less attention. Here we assess the relation between fatigue and regional WM-LL volumetric measures. 63 patients with RRMS participated in this study; mean age was 31.9 ± 8.1 years. Each patient provided demographic details and was scored on the expanded disability status scale (EDSS) and fatigue severity scale (FSS). VolBrain, a fully automated, operator-independent tool was used to assess WM-LL and whole brain volume. The patients were classified into three groups: no fatigue (FSS < 4), low to moderate fatigue (FSS ≥ 4 ≤ 5) and high fatigue (FSS > 5). 33.3% of patients had no significant fatigue, 25.4% had mild-to-moderate fatigue, and 41.3% had significant fatigue. Age, disease duration, relapses, and EDSS were positively correlated to fatigue severity (*P* = 0.034, 0.002, 0.009 and 0.001 respectively). Whole brain volume, total and regional WM-LL (juxtacortical, periventricular, infratentorial) were also correlated with fatigue severity. Ordinal regression analysis for fatigue severity showed EDSS and infratentorial lesion volume were the best predictors. In conclusion, EDSS and infratentorial lesion volume (cerebellar and brainstem) are the best predictors of fatigue severity.

## Introduction

Fatigue is one of the most important and disabling symptoms of multiple sclerosis (MS) patients^1^ and occurs in 80% of cases with relapsing remitting multiple sclerosis (RRMS). It is defined as difficulty initiating or sustaining voluntary activities and is accompanied by a feeling of disproportionate effort^2,3^. Fatigue has direct impact on the quality of life, and patient’s disability^4^. The precise mechanisms causing fatigue in MS patients are complex and poorly understood and may differ between patients. Advanced quantitative magnetic resonance imaging (MRI) techniques allow for objective assessment of disease pathology and have been used to characterize the pathophysiology of central fatigue in MS, but many questions remain regarding the anatomic brain correlate of fatigue.

Few papers have examined white matter lesion load (WM-LL) in relation to fatigue. Many early studies failed to identify an association between MS-related fatigue and either the total global extent of cerebral abnormalities or lesions in regional areas^5–8^. Several theories have been suggested for the lack of correlation between anatomical features and central fatigue. One study demonstrated that thalamic and cerebellar atrophy may be predictors of subsequent fatigue development^9^. Recently Khedr and their Colleague^10^ found that cerebral grey matter and thalamic volumes were negatively correlated with fatigue severity, but they did not measure WM-LL. Some previous studies reported fronto-parietal tract disruption and extensive white matter lesions in highly fatigued patients^11,12^.

The aim of the present was aimed to assess the relationship between fatigue and WM-LL and location and gray matter atrophy in a large sample of RRMS.

## Results

Sixty-three MS patients with a diagnosis of RRMS participated in the study. Table 1 includes demographic and clinical information. Mean age was 31.9 years, with an SD of 8.1. The average Expanded Disability Status Scale (EDSS), duration of the illness, and number of relapses were 3.4 (1.8), 51.3 months (47.7), and 3.4 (2.2), respectively. Mean time interval between disease onset and DMT was 32.1 months (40). Forty-two patients (66.7%) in our sample showed signs of fatigue, of which 25.4% had moderate fatigue and 41.3% had considerable fatigue.

**Table 1:** Demographic, clinical data and volumetric data of studied patients.

	Mean ± SD (Range) or number and %
Age years	31.9 ± 8.1 (16–47)
Sex (M/F) ratio	18/45 (28.6%/71.4%)
Marital status (single/married/divorced)	24/37/2 (38.1%/58.7%/3.2%)
Fatigue (No/Mild/significant)	21/16/26 (33.3%/25.4%/41.3%)
EDSS	3.4 ± 1.8 (0–7)
Disease duration (in months)	51.8 ± 47.7 (5–228)
Number of relapses	3.4 ± 2.3 (1–10)
Time to initiate DMT (in months)	32.1 ± 40.0 (0–210)
Whole brain Volume	1211.0 ± 84.7 (1032.8–1503.1)
Lesion count	28.1 ± 12.4 (2–57)
Lesion volume	18.7 ± 16.0 (0.1–55.5)
Juxtacortical lesion count	18.3 ± 10.1 (1–48)
Juxtacortical lesion volume	1.5 ± 1.4 (0.02–6.7)
Periventricular lesion count	7.9 ± 5.3 (1–33)
Periventricular lesion volume	16.1 ± 14.4 (0.07–48.8)
Infratentorial lesion count	0.8 ± 1.1 (0–3)
Infratentorial lesion volume	0.3 ± 0.5 (0–1.68)
Current DMT	
No treatment	5 (7.9%)
Interferon B	40 (63.5%)
Fingolimod	16 (25.4%)
Teriflunomide	1 (1.6%)
Rituximab	1 (1.6%)

Only 33.3% did not experience fatigue. Regarding current disease-modifying therapies (DMT), most patients were treated with interferon B (63.5%) followed by fingolimod (25.4%).

**Figure 1:**
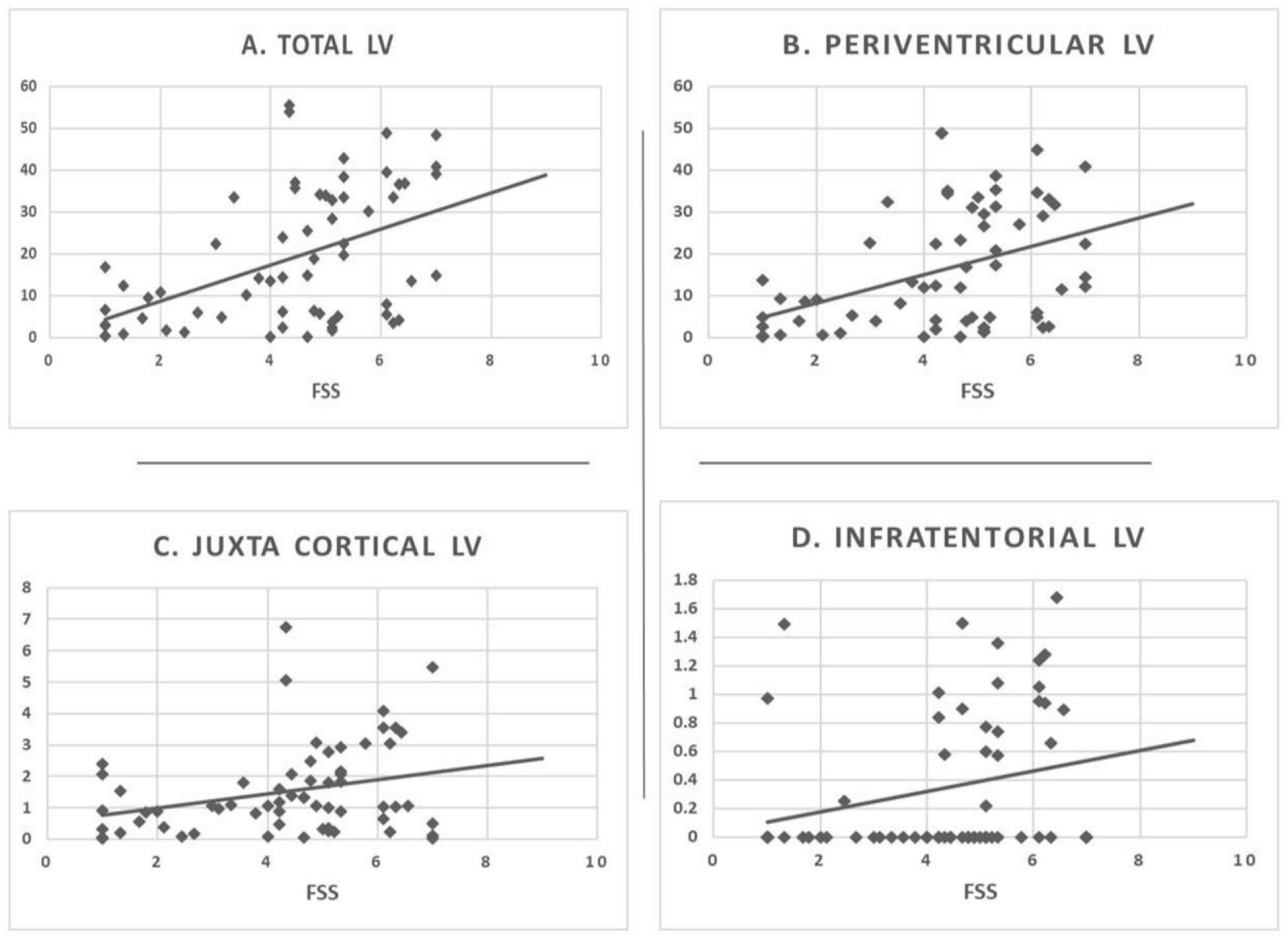
Scattered plot showing the relationship of FSS scores to total and regional lesion volumes. *LV* Lesion volume, *FSS* fatigue severity scale.

The Spearman correlations between study variables and fatigue level (no fatigue, mild to moderate fatigue, and high fatigue) are shown in Table 2. Positive correlations were found between age, disease duration, time to initiate DMT and relapse number (*p* = 0.034, 0.002, 0.028 and 0.009, respectively), while there was no association between gender and fatigue. There was also a significant positive correlation between EDSS and fatigue severity (*p* = 0.001). Significant positive associations between fatigue severity and both total and regional WM-LL were found. *P* values were 0.002 for total lesion volumes, 0.003 for periventricular lesion volume, 0.023 for juxtacortical lesion volume, and 0.003 for infratentorial lesion volume. This is also confirmed by using the raw data of FSS (Fig. 1).

**Table 2:** Correlation of fatigue levels (No fatigue/low to moderate fatigue/high fatigue) to clinical and volumetric data.

		Fatigue severity
Age	Spearman coefficient	0.256*
	Sig. (2-tailed)	0.034
Sex	Spearman coefficient	0.095
	Sig. (2-tailed)	0.192
Disease duration	Spearman coefficient	0.402**
	Sig. (2-tailed)	0.002
Number of relapses	Spearman coefficient	0.333**
	Sig. (2-tailed)	0.009
EDSS	Spearman coefficient	0.430**
	Sig. (2-tailed)	0.001
Time to initiate DMT	Spearman coefficient	0.271*
	Sig. (2-tailed)	0.028
Whole brain volume	Spearman coefficient	− 0.423-**
	Sig. (2-tailed)	0.002
Lesion count	Spearman coefficient	0.361**
	Sig. (2-tailed)	0.005
Lesion volume	Spearman coefficient	0.418**
	Sig. (2-tailed)	0.002
Juxtacortical lesion count	Spearman coefficient	0.351**
	Sig. (2-tailed)	0.006
Juxtacortical lesion volume	Spearman coefficient	0.287*
	Sig. (2-tailed)	0.023
Periventricular lesion count	Spearman coefficient	0.171
	Sig. (2-tailed)	0.106
Periventricular lesion volume	Spearman coefficient	0.382**
	Sig. (2-tailed)	0.003
Infratentorial lesion count	Spearman coefficient	0.411**
	Sig. (2-tailed)	0.002
Infratentorial lesion volume	Spearman coefficient	0.375**
	Sig. (2-tailed)	0.003

To investigate the influence of DMT on our research variables, we compared patients receiving interferon B to those receiving fingolimod in terms of the primary factors affecting fatigue in our data. An independent student t test revealed no statistically significant differences between the two groups in terms of age, disease duration, EDSS, or FSS scores. The group treated with Fingolimod showed a marginally significant increase in the number of relapses, with a *p*-value of 0.043. There were also no differences in total brain volume and total WM-LL. Table 3.

**Table 3:** Comparison between MS patients on interferon and fingolimod regarding main study variables.

	MS patients on interferon B n = 18 Mean ± SD	MS patients on fingolimod n = 25 Mean ± SD	*P*
Age	31.4 ± 7.6	33.8 ± 9.0	0.158
Disease duration	55.5 ± 51. 8	39.1 ± 30.0	0.129
Relapse number	3.1 ± 2.1	4.4 ± 2.5	0.043
EDSS	3.5 ± 1.7	3.7 ± 2.0	0.235
FSS	4.3 ± 1.6	4. 7 ± 2.1	0.192
Whole brain volume	1204.3 ± 76. 4	1203.9 ± 88.5	0.314
Total lesion volume	17.7 ± 15.4	25.5 ± 17.50	0.065

Table 4 compares patients with and without infratentorial lesions across various parameters. Age, disease duration, age at onset, EDSS, and time to start DMT show no significant differences between the groups. However, patients with infratentorial lesions have a significantly higher mean fatigue severity (FSS) compared to those without lesions with *p* value 0.030.

**Table 4:** Comparison between MS patients with and without infratentorial lesions regarding main study variables.

	Patients with no Infratentorial lesions n = 40 Mean ± SD	Patients with Infratentorial lesions n = 23 Mean ± SD	*P* value
Age	32.35 ± 7.957	31.22 ± 8.549	0.606
Disease duration	45.53 ± 45.282	61.57 ± 51.079	0.218
Age at onset	27.13 ± 7.690	25.52 ± 8.485	0.459
EDSS	3.350 ± 1.8404	3.609 ± 1.6442	0.568
Time to start DMT	30.03 ± 36.377	35.65 ± 46.240	0.619
FSS	3.9746 ± 1.85277	4.9404 ± 1.52622	0.030

Table 5 shows the results of multivariate logistic ordinal regression analysis (by forward stepwise method) using levels of Fatigue as the dependent variable. The model included disease duration, EDSS, time between onset to DMT, number of relapses, whole brain volume, juxta cortical lesion volume, periventricular lesion volume and infratentorial lesion volume. It was controlled for age, sex, and current DMT. Only EDSS and infratentorial lesion volume were identified as the best predictors, with *p*-values of 0.023 and 0.021, respectively.

**Table 5:** Logistic ordinal regression for fatigue severity groups using clinical data, volumetric and regional white matter lesion load data, after controlling for age, sex and current DMT drugs (forward stepwise method was applied).

	B	SE	Wald	df	Sig
EDSS	0.642	0.282	5.182	1	0.023
Infratentorial lesion volume	2.084	0.903	5.326	1	0.021

## Discussion

The current study examined the relation between total and regional white matter lesion load and fatigue in RRMS. Fatigue is difficult to measure as it is a totally subjective sensation. It includes symptoms of physical fatigue including sense of muscle weakness as well as mental fatigue characterized by difficult concentration, attention, thinking and poor memory^13^. Forty-two patients (66.7%) in our sample had fatigue (25.4% mild fatigue and 41.3% with significant fatigue). Previous studies have found a prevalence of MS-related fatigue of between 50 and 90%^14–16^. This large range may be attributable to discrepancies in the selection of research participants, the size of the study population, and the methodologies employed to measure the degree of fatigue.

The current study included individuals diagnosed with RRMS who had a mean EDSS of 3.4 ± 1.8 (ranging from 0 to 7) and an illness duration of 51.8 ± 47.7 months, indicating rapid disease progression. However, the mean time from symptom onset to the initiation of Disease-Modifying Therapies (DMTs) treatment was long, at 32.1 ± 40.0 months, and almost half of the patients had delayed diagnosis. This delay in diagnosis was consistent with our previous study^17^, which attributed the delay to a higher rate of initial non-motor presentation, non-neurological consultations, prior misdiagnoses, and reluctance of relatives to seek medical advice in developing countries like Egypt. Delayed diagnosis leads to delayed DMT initiation, resulting in an increased frequency of relapses and rapid disease progression.

The present study supported the results of the previous study^10^ and found that fatigue severity increased with age, illness duration, and EDSS score, which may be related to progressive brain atrophy in MS patients with age and time, contributing to fatigue. A strong positive association was found between the EDSS and FSS, consistent with previous research^18–20^. In addition, a significant connection was identified between brain atrophy and fatigue, supporting previous research^8,21–23^.

The FSS scale is widely used in therapeutic settings to track changes in physical fatigue over time. In this study, we focused solely on the physical aspect of fatigue and therefore chose the FSS as our assessment tool. While some studies have excluded patients with FSS scores of 4 or 5 and categorized patients as either having mild fatigue (FSS score < 4) or significant fatigue (FSS score > 5), our study included all patients and classified them into three categories.^23,24^.

The categorization of severity into three groups provides a more nuanced and accurate assessment of fatigue. This should improve the ability of researchers to detect changes in fatigue over time and evaluate the effectiveness of interventions. It means, for example that it is possible to identify an intervention that significantly reduces fatigue in individuals with moderate fatigue, which may not be detected if only two classifications are used (fatigued and not fatigued). Alternatively, patients with severe fatigue may require more intensive interventions than those with mild or moderate fatigue. This tripartite classification is a compromise between using the raw data FSS scores (e.g. Khedr et al.^10^ a dual classification, which simplifies the analysis and presentation of results.

Khedr and their colleagues^10^ found that fatigued MS patients had greater reduction in total brain volume, cerebral grey matter, and volumes of brain stem, thalamus, and caudate compared to non-fatigued patients. Regression analysis indicated that thalamic and brain stem atrophy are the best predictors of fatigue. Our study complements this by showing a significant relationship between infratentorial lesion volume and fatigue. Therefore, the best predictors of fatigue from both studies are thalamic and brain stem atrophy, as well as WM-LL of the infratentorial region (cerebellum and brain stem).

Our study differs from the findings of Weier et al.’s study^25^, which examined the correlation between cerebellar volumes, clinical cerebellar signs, cognitive functioning, and fatigue in MS and did not identify a significant association between cerebellar abnormalities and fatigue. However, it is worth noting that the two studies utilized different methods of assessing fatigue. The current study employed the FSS, whereas Weier et al., used a brief neuropsychological assessment that included a fatigue testing component. Furthermore, the two studies had different focuses; our study investigated predictors of fatigue severity, while Weier et al., explored the relationship between cerebellar abnormalities and cognitive impairment in MS.

Several previous structural MRI studies of fatigue explored the connection between fatigue and WM lesions in MS. Some of these identified a significant connection between fatigue and total brain WM lesion load^11,23^, or regional WM-LL^26–28^ whereas other using a similar methodology were unable to do so^22,29,30^. The variable sensitivity of the methods employed to identify damage in normal-appearing white matter (NAWM), and variation in the clinical populations included in these studies may account for such discrepancies.

We failed to identify a significant correlation between the current DMT and disability or fatigue levels. Due to the limited sample size, we only compared patients on interferon B to those on fingolimod. The absence of a significant difference between DMTs could be partially related to the study design as the current study was cross-sectional. However recent papers have reported similar results with no significant differences between the two drugs for EDSS, cognition, or fatigue^31^. Again, they had a small sample size and did not follow patients longitudinally. Long-term usage of DMT has been shown to have a more beneficial impact^32^. Cohen et al.^33^ found fingolimod to be more effective than interferon B in slowing disease progression and reducing brain volume loss.

Our research revealed a significant relationship between the volume of infratentorial lesions and fatigue. After adjusting for age, gender, and current DMT, this association was maintained, but at a reduced strength. As far as we know this has not been reported in many previous studies. A recent study found that selective cerebellar pathology could potentially play a role in the development of fatigue and depression in RRMS, which in turn may exacerbate disability in these individuals.^34^ Since the cerebellum plays a critical role in action control and motor learning, and its non-motor activities are gaining recognition^35^, it may well contribute to the sense of fatigue.

On the other hand, it has been speculated that the ascending reticular activating system (ARAS) has a role in the development of fatigue^36^. The neocortex, the thalamus, and the hypothalamus are all connected to the ARAS, which originates in the brainstem^37^. Some researchers hypothesized that lesions in the tegmentum of the upper pons and midbrain, where the ARAS is thought to begin, would have knock-on effects on other brain regions^38^.

To the best of our knowledge, limited research has been conducted to elucidate the structural alterations in the brain associated with fatigue in multiple sclerosis (MS) patients. In a prior study by Khedr et al., we quantified brain volumetric changes in fatigued and non-fatigued individuals with relapsing–remitting MS (RRMS) and identified that atrophy of the thalamus and brain stem exhibited the strongest predictive value for fatigue^10^. Another study by Riccitelli et al. employed a comprehensive voxel-wise analysis of the entire brain to examine the regional distribution patterns of lesions and revealed atrophy in the left central sulcus and precentral gyrus (primary sensory motor area) among fatigued patients^39^. Moreover, previous research by Cruz Gómez et al. and Damasceno et al., has established a correlation between cerebellar volume reduction, brain stem volume loss, and fatigue in MS^40,41^. In the present study, we specifically observed a significant association between infratentorial lesion volume load and fatigue, highlighting infratentorial lesion load as the most reliable predictor of fatigue. This could be explained as cortical gray matter atrophy, as well as cerebellar and brain stem lesions, which may impede efficient motor and sensory information processing between cortical areas and cerebellar/brain stem regions, ultimately leading to the manifestation of fatigue^40^.

## Limitations of the study

The most important limitations of the current study were the small sample size and its cross-sectional nature. To replicate the findings, a longitudinal follow-up of MS patients with larger sample size would be required, particularly to evaluate the influence of various DMTs on brain atrophy measures. Lack of screening for depression, which may or may not have had a role in the development of fatigue, is another limitation.

## Conclusion

The present results demonstrate the importance of EDSS and infratentorial lesion volume (cerebellar and brainstem) as predictors of fatigue severity in RRMS.

## Methods

### Participants

Out of 80 cases of RRMS, 63 individuals were included in this study and 17 patients were excluded from the analysis, either due to MRI artifact or rejection by the LesionBrain tool due to segmentation errors. All patients included in the study were diagnosed with RRMS using the 2017 McDonald diagnostic criteria, and presented to MS clinics in Assiut, Qena, and Armant Hospitals in Egypt. Patient recruitment took place over a period of 1.5 years, from October 2020 to April 2022.

*Inclusion criteria*: Age 18–50 years of either sex

*Exclusion criteria*: Patients who had a relapse or had used steroids in the previous 30 days. Patients with any chronic medical illnesses that cause fatigue, as well as those taking psychoactive medication or drugs that alleviate fatigue were also excluded.

All patients had a thorough medical and neurological history and examination.

The institutional review board of the Faculty of Medicine at Assiut University gave ethical permission for the study (17,300,884) and conducted in accordance with the provisions of the Declaration of Helsinki. Written Informed consent was obtained from all participants before enrolment.

### Study procedures

Each patient provided:

- Demographic and clinical data; including age, age of onset, sex, duration of illness, and number of relapses, types of DMTs.
- EDSS^42^ was calculated and data about current DMT were collected.
- Fatigue assessment

The degree of fatigue was determined using the Arabic-validated version of the fatigue severity scale^43,44^. There are a total of 9 elements on the scale, and the patient may choose a score between 1 and 7 for each, where 1 indicates strong disagreement and 7 strong agreements. The average score is then determined. Patients with an average score of less than 4 were classified as having no fatigue. Patients with a score between 4 and 5 were classified as moderate fatigue. Patients with a score higher than 5 were classified as having severe fatigue.

Using three FSS groups instead of only two groups (fatigued and not fatigued) in MS research provides a more nuanced understanding of the severity of fatigue experienced by individuals with MS. By dividing the FSS into three groups into no fatigue, mild to moderate fatigue, and high fatigue categories, researchers gain a better understanding of the impact of fatigue on daily activities and quality of life. This information can be used to tailor interventions to the specific needs of individuals with MS who experience varying levels of fatigue severity.

- Magnetic resonance Imaging (MRI):

### Machine specifications

The study was conducted at three different centers in upper Egypt, all of which were equipped with a Philips Achieva 1.5 T MRI machine. MRI acquisition was done with the same methodology. A T1-weighted spin-echo axial picture (TR/TE = 600/15 ms, flip angle = 90°, FOV (frequency/phase) = 220/75, acquisition matrix size = 256 256, voxel size = 1 1 3 mm) and a T2-weighted FLAIR (fluid-attenuated inversion recovery) image (TR/TE = 9000/110 ms, inversion time = 2500 ms, flip angle = 130◦, FOV (frequency/ phase) = 220/75, acquisition matrix size = 320 × 168, voxel size = 1 × 1 × 3 mm) were collected, and they covered the whole brain so that we could calculate the volumes of the brain and any lesions present.

The volumetric data was obtained through the acquisition of magnetic resonance imaging (MRI) data, including T1 and FLAIR sequences. These sequences were converted from Digital Imaging and Communications in Medicine (DICOM) files to two compressed, anonymized Neuroimaging Informatics Technology Initiative (NIfTI) files—one for T1 and the other for FLAIR—which were used for subsequent analyses.

- Brain volume and lesion load assessment

LesionBrain 1.0 is an accessible web-based application that has been designed specifically for the segmentation of white matter lesions^45^. This software has been successfully integrated into the volBrain platform (https://volbrain.upv.es/)^46^, providing a comprehensive tool for the accurate and efficient detection of lesions. The processing pipeline is comprised of several crucial steps, including image normalization and registration, structure segmentation to identify the intracranial cavity, brainstem, cerebellum, and lateral ventricles, and candidate mapping to identify areas that may potentially contain lesions. The lesions are then segmented using a voxel-wise method, which employs a three-step approach comprising patch-based multimodal segmentation, patch-based regularization of the generated lesion probability map, and an ensemble of shallow neural networks to rectify any erroneous patches, thereby minimizing false positives. Importantly, this pipeline has been rigorously evaluated using the MSSEG MICCAI Challenge 2016 dataset and has demonstrated good performance, with a mean Dice coefficient of 0.66^47^. The whole brain volume, total numbers of lesions, the absolute volume of all lesions either total or regional (in cubic centimeters) were all measured.

### Analysis

All statistical analyses were conducted using SPSS for Windows version 26. Descriptive statistics were performed to summarize the data, with numerical data presented as mean and standard deviation (SD) and categorical data presented as frequencies and percentages. The normality of the variables was assessed using the Shapiro–Wilks and Kolmogorov–Smirnov tests. The study patients were categorized into three ordinal groups based on their scores on the Fatigue Severity Scale (FSS) as follows: no fatigue, moderate fatigue, and severe fatigue. Spearman correlation coefficient was used to assess the association between all clinical and volumetric data and fatigue severity. Multivariate logistic ordinal regression analysis was performed to examine the predictability of research factors for fatigue, with the dependent variable being the ordinal fatigue severity grouping. The volumetric and clinical data were tested separately, with both controlled for age, sex, and current Disease-Modifying Therapy (DMT). To address multiple comparisons, we applied the false discovery rate (FDR) correction using MATLAB software version R2020a. The original P-values were replaced with the adjusted ones obtained from MATLAB. The statistical significance was set at *P* < 0.05 for all tests.

## Data Availability

Data can be made available to qualified investigators upon reasonable request to the corresponding author.

## Abbreviations

MS: Multiple sclerosis
RRMS: Relapsing remitting multiple sclerosis
WM-LL: White matter lesion load
FSS: Fatigue severity scale scoring
MRI: Magnetic resonance imaging
NAWM: Normal-appearing white matter
DMTs: Disease-modifying therapies
EDSS: The expanded disability status scale
